# Berberine Impairs the Survival of Triple Negative Breast Cancer Cells: Cellular and Molecular Analyses

**DOI:** 10.3390/molecules25030506

**Published:** 2020-01-24

**Authors:** Lamyae El Khalki, Virginie Maire, Thierry Dubois, Abdelmajid Zyad

**Affiliations:** 1 Team of Experimental Oncology and Natural Substances, Cellular and Molecular Immunopharmacology, Faculty of Sciences and Techniques, Sultan Moulay Slimane University, Mailbox 523, 23000 Beni Mellal, Morocco; elkhalki.lamyae@gmail.com; 2 Breast Cancer Biology Group, Translational Research Department, Institut Curie-PSL Research University, 75005 Paris, France; virginie.maire@curie.fr (V.M.); thierry.dubois@curie.fr (T.D.)

**Keywords:** apoptosis, berberine, cell cycle, cytotoxicity, triple negative breast cancer

## Abstract

Triple negative breast cancer (TNBC) is an aggressive breast cancer subtype. Non-available targeted therapy for TNBC represents its biggest treatment challenge. Thus, finding new promising effective drugs is urgently needed. In the present study, we investigated how berberine, a natural isoquinoline, impairs the survival of TNBC cells in both cellular and molecular levels. Our experimental model was based on the use of eight TNBC cell lines: MDA-MB-468, MDA-MB-231, HCC70, HCC38, HCC1937, HCC1143, BT-20, and BT-549. Berberine was cytotoxic against all treated TNBC cell lines. The most sensitive cell lines were HCC70 (IC_50_ = 0.19 µM), BT-20 (IC_50_ = 0.23 µM) and MDA-MB-468 (IC_50_ = 0.48 µM). Using flow cytometry techniques, berberine, at 0.5 and 1 µM for 120 and 144 h, not only induced cell cycle arrest, at G1 and/or G2/M phases, but it also triggered significant apoptosis. At the molecular level, these results are consistent with the expression of their related proteins using Western blot assays. Interestingly, while berberine was cytotoxic against TNBC cells, it had no effect on the viability of normal human breast cells MCF10A cultured in a 3D matrigel model. These results suggest that berberine may be a good potential candidate for TNBC drug development.

## 1. Introduction

Breast cancer continues to be the most prevalent cancer in the vast majority of countries globally. According to Globocan 2018, new breast cancer cases are estimated by 11.6% worldwide in 2018, and approximately 12% of these cases are triple negative breast cancer (TNBC) [1,2], which is an aggressive breast cancer subtype with no targeted therapy due to the absence of estrogen and progesterone hormone receptors with the lack of human epidermal growth factor receptor-2/Neu over-expression [2]. Since targeted therapy is not yet available for TNBC patients, cytotoxic chemotherapy is the only available way for their treatment. Unfortunately, chemotherapy does not only kill cancer cells but also causes normal growing cells to die, leading to severe side effects [3]. Thus, new treatments are urgently required to improve the survival rate and patients’ life quality.

Berberine (PubChem CID: 2353) [4], is a natural isoquinoline alkaloid compound isolated from the stems and roots of several plants such as *Berberis vulgaris*, *Berberis aristata*, *Berberis asiatica, Coptidis rhizome, Coptidis chinensis, Coptidis japonica, Mahonia aquifolium,* and *Mahonia beale* [5,6]. It exerts several pharmacological activities such as antiplatelet, antibacterial, anti-inflammatory, immunomodulatory, anti-oxidative, neuroprotective, anti-diabetic, and hypolipidemic [6,7].

Several preclinical studies have reported the anticancer effect of berberine where it exhibited its inhibitory effects on a variety of tumours such as hepatoma, leukemia, breast, lung, colon, ovarian and cervical cancer cells through apoptosis induction and cell cycle arrest, inhibition of migration and invasion, reduction of the expression of VEGF mRNA and inhibition of angiogenesis [8].

Here, we aimed to explore the mechanisms of berberine’s effect on the behavior of several TNBC cell lines, such as proliferation, colony formation, cell cycle progression, DNA damage, and apoptosis in both cellular and molecular levels. Furthermore, and as long as the main problem of chemotherapy regimen is systemic toxicity, we investigated the effect of berberine on the viability of normal human breast epithelial cells.

## 2. Results

### 2.1. Berberine Inhibits Proliferation of Triple Negative Breast Cancer (TNBC) Cells

**Figure 1:**
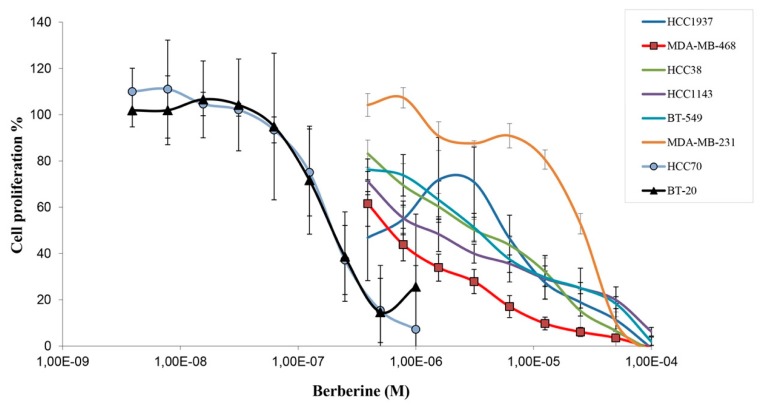
Effect of berberine’s treatment on triple negative breast cancer (TNBC) cell proliferation. TNBC cell lines were seeded and treated with berberine. The cell viability was measured by MTT assay. ± Standard Deviation of three independent experiments carried out in triplicate.

Screening berberine’s anti-proliferative activity on 8 different TNBC cell lines, through MTT assay, showed that berberine inhibited their growth in a dose dependent manner, with IC_50_ values ranging from 0.19 µM to 16.7 μM (Figure 1 and Table 1). According to IC_50_ values and the curve shapes of the treated cell lines, we noticed that the cells have different responses towards the treatment depending on the doses of berberine, when HCC70 (IC_50_ = 0.19 µM), BT-20 (IC_50_ = 0.23 µM) and MDA-MB-468 cells (IC_50_ = 0.48 µM) were found to be the most sensitive ones to berberine treatment and inversely, MDA-MB-231 was the most resistant one (IC_50_ = 16.7 µM) among all the treated cell lines (Figure 1 and Table 1).

**Figure 2:**
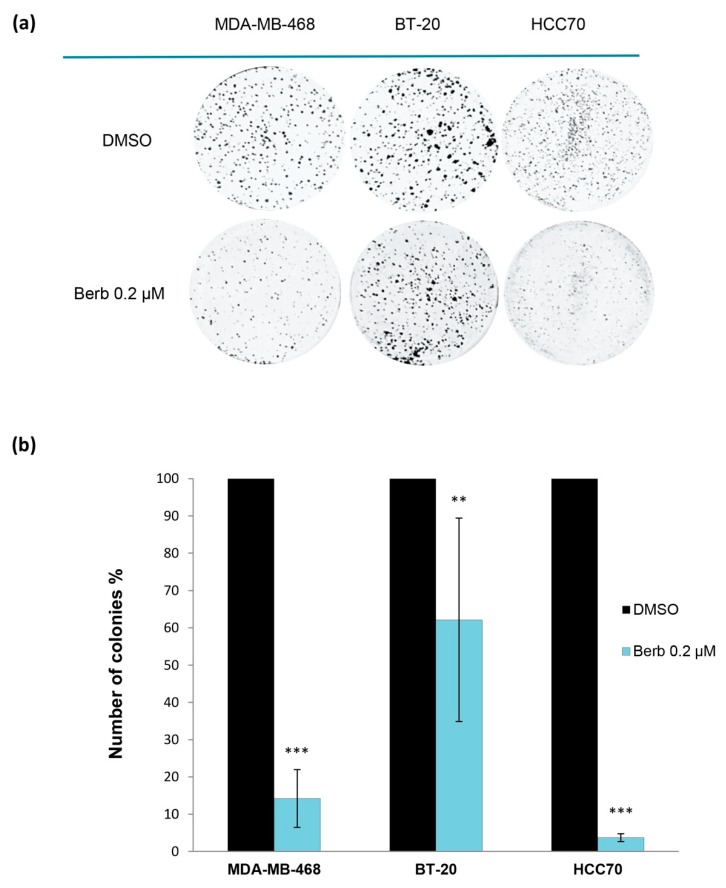
Berberine’s inhibition of TNBC cell lines colony formation. (**a**) Pictures of wells containing colonies of berberine-treated TNBC cell lines. (**b**) Number of colonies % vs control (DMSO) of each treated cell line. TNBC cell lines: MDA-MB-468, BT-20 and HCC70. Berb—Berberine. ±Standard Deviation of three independent experiments done in triplicate, * *p* < 0.05, ** *p* < 0.01 and *** *p* < 0.001 compared to DMSO.

Further to establish the inhibitory role of berberine on transforming properties of cancer cells, we performed the 2D-clonogenic assay. When compared to DMSO treated cells, results showed that berberine significantly reduced colony formation of MDA-MB-468 (*p* < 0.001), BT-20 (*p* < 0.01) and HCC70 (*p* < 0.001) at 0.2 µM, indicating a potent cell growth inhibition (Figure 2).

### 2.2. Berberine Differentially Affects TNBC Cell Cycle Progression

**Figure 3:**
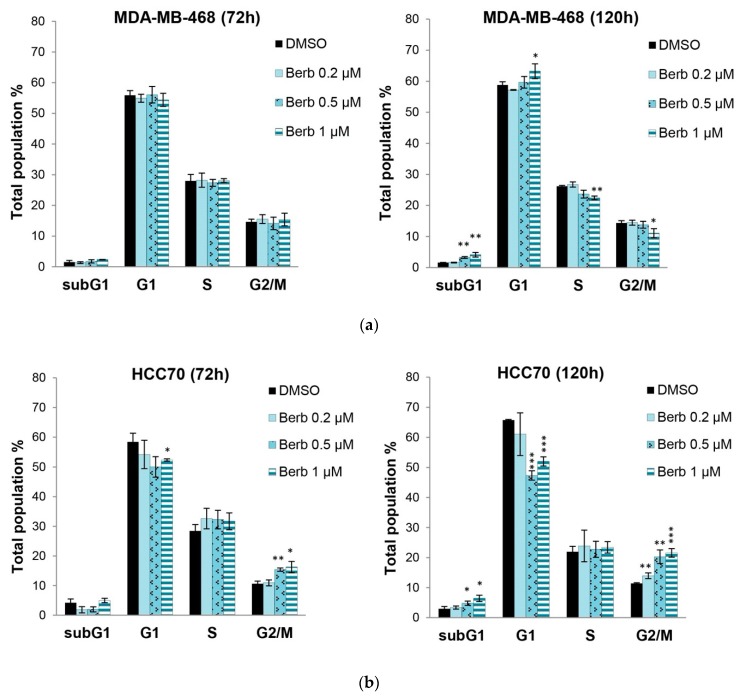

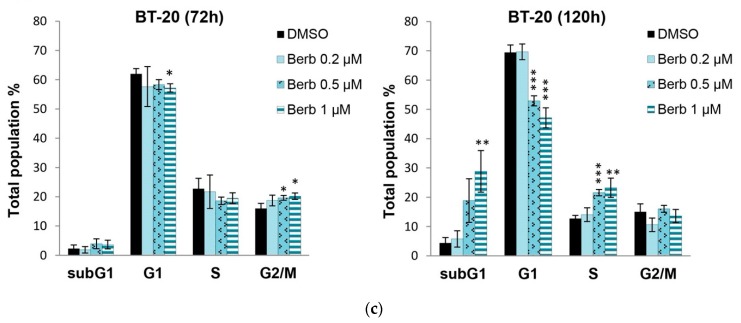
Berberine’s effects on TNBC cell cycle progression depending on its doses (µM) 0.2, 0.5 and 1, and time for 72 h and 120 h. Total population % of cell cycle phases (G1, S, G2/M and SubG1) ± SD of three independent experiments, * *p* < 0.05, ** *p* < 0.01 and *** *p* < 0.001 compared to DMSO. (**a**) MDA-MB-468, (**b**) HCC70 and (**c**) BT-20. Berb—Berberine.

**Figure 4:**
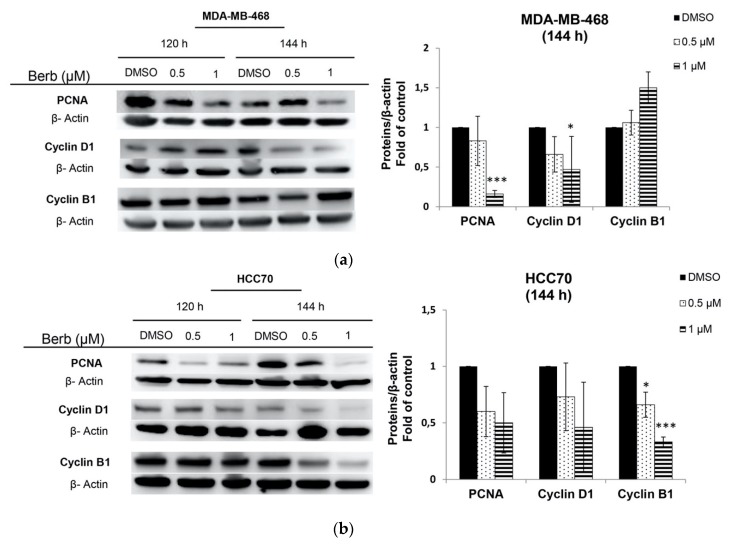

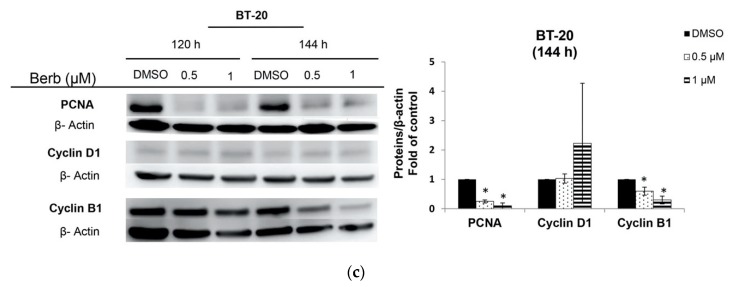
Representative Western blot results, of the expression of cell cycle related proteins PCNA, Cyclin D1 and Cyclin B1 of (**a**) MDA-MB-468, (**b**) HCC70 and (**c**) BT-20 treated with berberine at 0.5 and 1 µM for 120 h and 144 h. Results of three independent experiments ± SD, * *p* < 0.05, ** *p* < 0.01 and *** *p* < 0.001 compared to DMSO, Berb—Berberine.

Since berberine inhibited cell proliferation, we further studied the role of this molecule on cell cycle progression in MDA-MB-468, HCC70 and BT-20 cells by flow cytometry. Results showed that cells had different responses towards berberine’s treatment depending on the cell line type (Figure 3). Berberine had no significant effect on MDA-MB-468 cell cycle at 72 h of the treatment. However, it significantly (*p* < 0.05) induced G1 phase arrest in MDA-MB-468 cells at 1 µM and at 120 h, in comparison to DMSO treated control cells (Figure 3a). The figure shows a significant increase in the percentage of cells in G1 phase (*p* < 0.05) with a concomitant significant decrease in the percentage of cells in S (*p* < 0.01) and G2/M (*p* < 0.05) phases, suggesting the role of berberine in inhibiting the entry into S phase of MDA-MB-468 cells (Figure 3a). This effect correlated with the changes in the amount of cell cycle proteins cyclin D1 and PCNA, at 144 h and not at 120 h (Figure 4a). Western blotting analysis showed a significant decrease in the amount of cyclin D1 (*p* < 0.05) and PCNA (*p* < 0.001) proteins after 144 h in MDA-MB-468 treated cells (Figure 4a).

However, in the case of HCC70 and BT-20 cells and at 120 h of the treatment, berberine at 0.5 and 1 µM significantly (*p* < 0.001) decreased their G1 phases with a simultaneous significant (*p* < 0.001) increase in G2/M phase of HCC70 cell line and a significant (*p* < 0.001) increase in S phase in BT-20 cells, compared to DMSO treated cells, as it is shown in Figure 3b,c. These results correlated with the Western blot results at 144 h, whereby cyclin B1 was significantly down-regulated in both cells, BT-20 (*p* < 0.05), and HCC70 (*p* < 0.001), with a significant (*p* < 0.05) decrease in PCNA expression in only BT-20 cell line, in comparison to DMSO treated cells (Figure 4b,c).

On the other hand, after 120 h of the treatment, we noticed a significant manifestation of the cell death shown by the SubG1 phase increase in all the three tested cell lines MDA-MB-468 (*p* < 0.01), HCC70 (*p* < 0.05) and BT-20 (*p* < 0.01) in comparison to DMSO treated control cells (Figure 3).

### 2.3. Berberine Induces DNA Damage in TNBC Cell Lines

**Figure 5:**
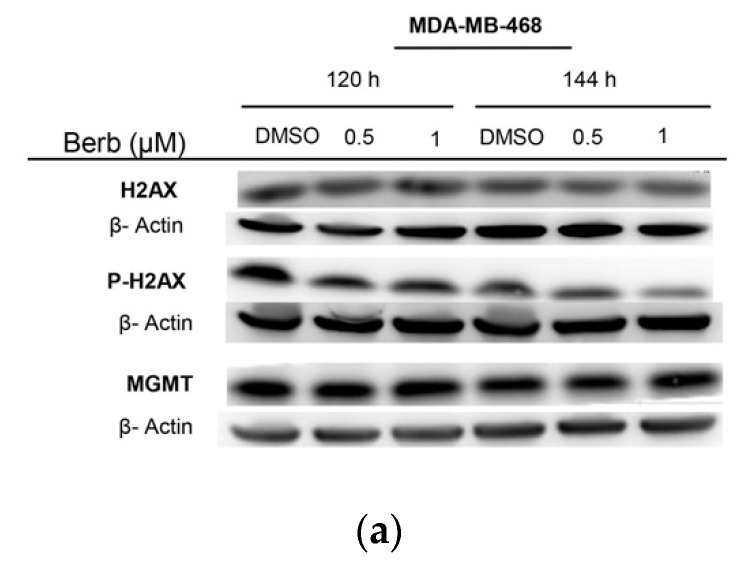

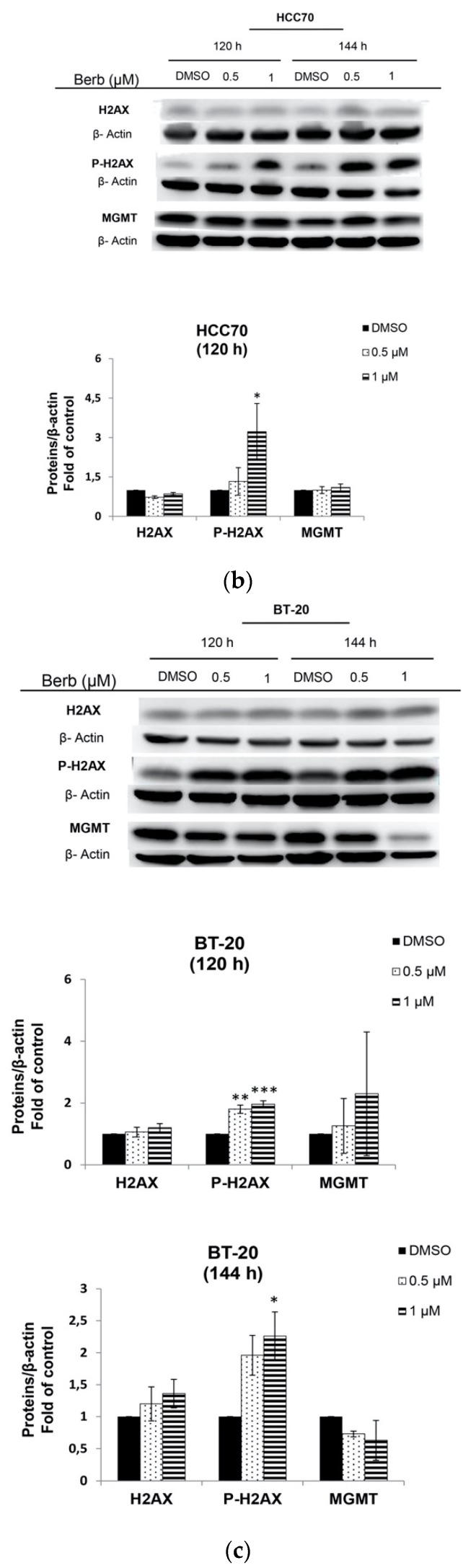
The effects of berberine treatment on the phosphorylation of H2AX and the expression of MGMT proteins of TNBC cell lines (**a**) MDA-MB-468, (**b**) HCC70 and (**c**) BT-20 at 120 h and 144 h. Representative Western blot results of three independent experiments. Berberine doses: 0.5 and 1 µM. ± SD, * *p* < 0.05, ** *p* < 0.01 and *** *p* < 0.001 compared to DMSO. Berb—Berberine.

Compared to DMSO treated cells, Western blot results showed that BT-20-berberine treated cells exhibited a significant (*p* < 0.001) increase in the phosphorylation of H2AX, both at 120 h and 144 h of the treatment, same with HCC70 cells that showed a significant (*p* < 0.05) dose dependent increase of H2AX phosphorylation at 120 h of the treatment (Figure 5b,c). These results indicate that those cells may be experiencing genotoxic stress under berberine treatment. On the other hand, berberine had no significant effect on H2AX phosphorylation and the expression of MGMT protein in MDA-MB-468 cells (Figure 5a).

### 2.4. Berberine Induced Apoptosis on TNBC Cell Lines in a Dose and Time Dependent Manner

**Figure 6:**
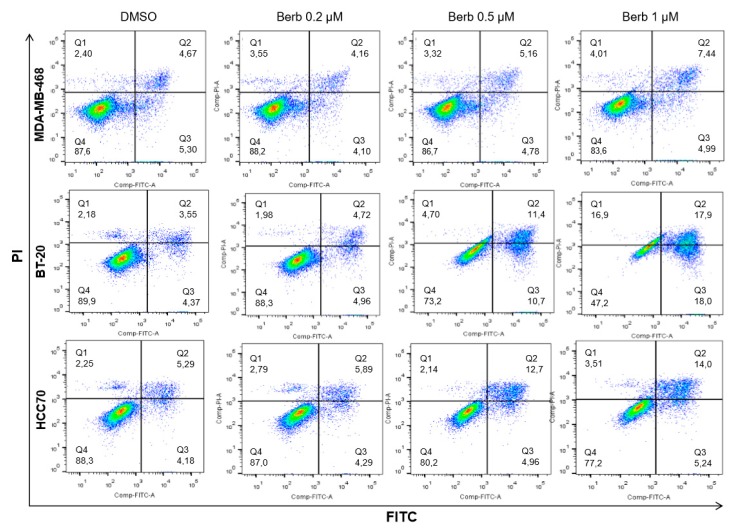
Annexin-V FITC and PI staining on TNBC cell lines: MDA-MB-468, BT-20 and HCC70 following the treatment with berberine (µM) 0.2, 0.5 and 1, at 144h. Q1: PI+/FITC-; Q2: PI+/FITC+; Q3: PI-/FITC+; Q4: PI-/FITC-. Berb—Berberine.

**Figure 7:**
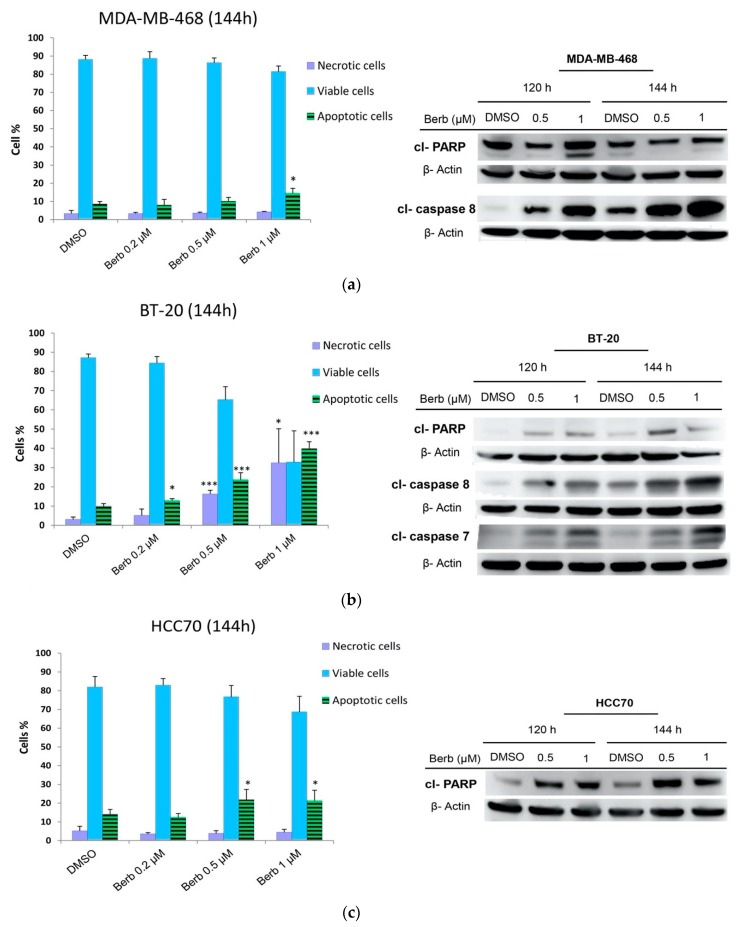
Berberine induced apoptosis in a dose and time dependent manner. Annexin-V and PI staining after 144 h of the treatment with berberine at 0.2, 0.5 and 1 µM. Western blot results of three independent experiments showing apoptosis related proteins expression upon berberine’s treatment at 120 h and 144 h. (**a**) MDA-MB-468, (**b**) BT-20, and (**c**) HCC70. ±SD of three independent experiments, * *p* < 0.05, ** *p* < 0.01 and *** *p* < 0.001 compared to DMSO. Berb—Berberine.

**Figure 8:**
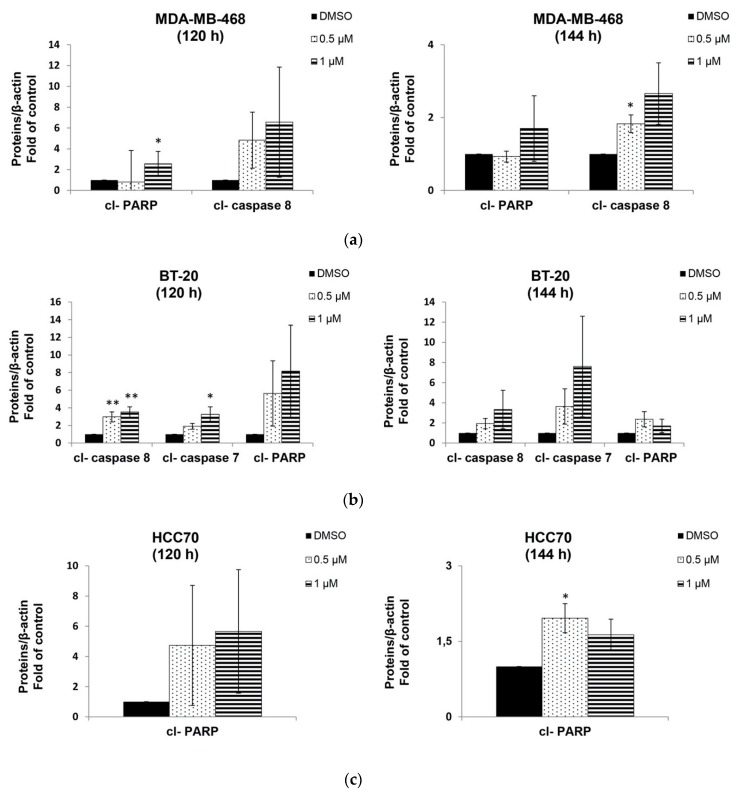
Fold of control of the western blot apoptosis related proteins expression upon berberine’s treatment at 120 h and 144 h: cl-PARP, cl-caspase 7 and cl-caspase 8. (**a**) MDA-MB-468, (**b**) BT-20 and (**c**) HCC70. ±SD of three independent experiments, * *p* < 0.05, ** *p* < 0.01 and *** *p* < 0.001 compared to DMSO. Berb—Berberine.

Cell cycle results already showed that SubG1 phases of the three tested cell lines (MDA-MB-468, BT-20 and HCC70) significantly increased after 120 h of the treatment with berberine, as compared to DMSO treated control cells (Figure 3). To confirm that berberine-induced loss of the proliferation capacity of TNBC cells was associated with the induction of apoptosis, MDA-MB-468, BT-20, and HCC70 were treated with berberine at 0.2, 0.5, and 1 µM and incubated for 120 h and 144 h. Afterwards, apoptotic cells were counted using Annexin-V and PI staining by flow cytometry (Figure 6 and Figure 7). Revelation of the apoptotic marker’s expression: cl-PARP, cl-caspase 8, and cl-caspase 7 by Western blot are shown in Figure 7 and Figure 8.

Berberine did not show a significant apoptosis neither in MDA-MB-468 nor in HCC70, but only in BT-20 treated cells at 120 h of the treatment (data not shown). However, Annexin-V staining results indicated that, at 144 h, berberine triggered apoptosis in all TNBC tested cells differently depending on the dose and the cell line (Figure 6 and Figure 7). MDA-MB-468 (*p* < 0.05), BT-20 (*p* < 0.001) and HCC70 (*p* < 0.05) cells exhibited a significant increase in the apoptotic populations compared to DMSO control cells (Figure 7). These results correlated with the findings of Western blot on the cleavage of PARP, caspase-7 and caspase-8 depending on the dose, time and the cell line (Figure 8). We noticed that berberine significantly induced the cleavage of PARP (*p* < 0.05) at 120 h of the treatment and the cleavage of caspase-8 proteins (*p* < 0.05) lately after 144 h of berberine’s treatment in MDA-MB-468 cells (Figure 8a). BT-20 cells exhibited a significant cleavage of caspase-7 (*p* < 0.05) and caspase-8 (*p* < 0.01) earlier at 120 h of the treatment (Figure 8b). However, in HCC70 cells, we only noticed a significant (*p* < 0.05) increase in the cleavage of PARP protein upon 144 h of berberine’s treatment (Figure 8c). The cleavage of PARP, caspase-7 and caspase-8 indicates the occurrence of apoptosis [9].

These findings demonstrate that berberine engender apoptotic cell death in TNBC cell lines through the cleavage of PARP, caspase-7, and caspase-8 proteins, all depending on the time, dose, and the cell line.

### 2.5. Berberine Does not Affect the Viability of “Normal” Human Breast MCF-10A Cells

**Figure 9:**
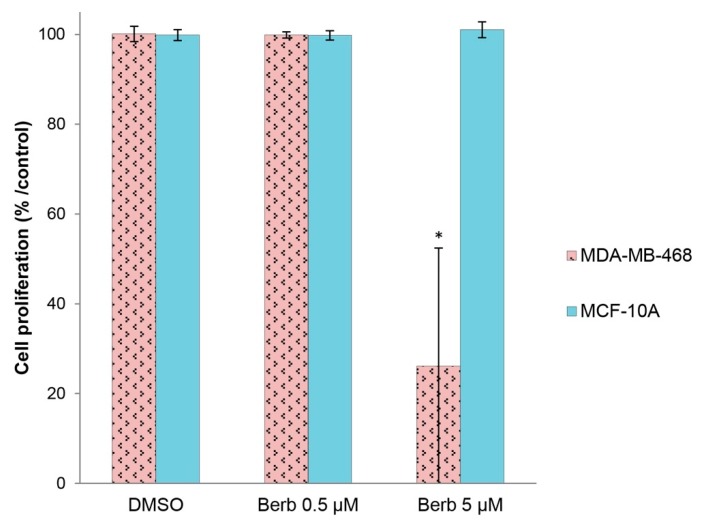
The effect of berberine on the viability of MDA-MB-468 TNBC malignant cells and MCF-10A normal Human epithelial breast cells cultured in matrigel 3D culture. Berberine’s doses: 0.5 and 5 µM. ±SD of three independent experiments, * *p* < 0.05, ** *p* < 0.01 and *** *p* < 0.001 compared to DMSO. Berb—Berberine.

Berberine was tested on MDA-MB-468 cell line grown in a more physiological context, in matrigel 3D culture [10]. Under these conditions, non-transformed mammary epithelial cell lines, such as MCF-10A, recapitulate epithelial morphogenesis by forming acinar structures within 10 days of culture, and then stop to grow, whereas cancer cells, such as MDA-MB-468, exhibit disorganized structures and continue to proliferate [11]. Berberine at 5 µM when added to these structures once formed, was significantly (*p* < 0.05) cytotoxic on malignant MDA-MB-468 cells while it had no significant effect on normal Human MCF-10A cells at the same dose of berberine (5 µM) (Figure 9). On the other hand, we didn’t notice any effect on MDA-MB-468 cells when treated by 0.5 µM of berberine, and this may be related to the fact that berberine does not penetrate well into matrigel and that the concentration of the inhibitor reaching the cells is lower.

## 3. Discussion

A total of 10%–20% of newly diagnosed breast cancers are TNBC [12]. Despite recent advances in diagnosis and treatment, patients with TNBC have been shown to have the highest rate of recurrence within the first five years after diagnosis compared with hormone receptor-positive (HR+) and HER-2/neu receptor-positive (HER2+) breast cancer patients [12]. Thus, underlying molecular mechanisms and new therapeutic strategies are urgently required.

To maintain tissue homeostasis, cell proliferation and death must be regulated. Many studies suggest that the regulation of the process of the cell cycle progression and programmed cell death may be achieved by controlling a set of factors such as cell cycle and apoptosis proteins [13].

Deregulated cell proliferation and inhibition of apoptosis are the main causes of all tumour development, they present two obvious targets for therapeutic intervention: (i) Interfering with the process of DNA synthesis and cell division. (ii) Targeting the suppressors of apoptosis in tumor cells [14]. In this paper, berberine showed an interesting inhibitory effect of the proliferation of several TNBC cell lines (BT-20, HCC70, MDA-MB-468, MDA-MB-231, HCC1143, BT-549, HCC38 and HCC1937) with an IC_50_ ranging from 0.19 µM to 16.7 µM; with BT-20, HCC70 and MDA-MB-468 being the most sensitive ones and BT-549 and MDA-MB-231 being the most resistant ones to berberine treatment. In fact, recent studies have found that berberine inhibits TNBC cell growth and metastasis [15,16,17,18,19,20].

We continued our study on the three most sensitive cell lines (MDA-MB-468, HCC70 and BT-20) to first explore berberine’s antiproliferative effect on their cell cycle progression. Interestingly, we found that berberine blocked cell proliferation in G2/M phases in both MDA-MB-468 and HCC70 cells, and blocked cell proliferation in S phase in BT-20. Interestingly, berberine decreased the expression of PCNA in MDA-MB-468 and BT-20, a proliferating cell nuclear antigen, the concentration of which changes during the cell cycle through its involvement in DNA replication and repair [21].

Mitotic events regulation is linked to the control of the activity of cyclin B1 protein to make cells enter mitosis, skip mitosis or arrest at G2 phase [22]. In our results, we found that cyclin B1 protein was also expressed at lower levels after berberine treatment in BT-20 and HCC70 causing G2/M phase arrest in HCC70. Previous findings report that berberine inhibited the expression of cyclin B1 to induce G2/M phase arrest in numerous cancer types [23,24].

Remarkably, berberine-treated (1 µM) MDA-MB-468 cells showed a significant increase in G1 phase population with a simultaneous significant decrease in S and G2/M phases at 120 h of the treatment. In all known cell cycle proteins, cyclin D1 was the most critical checkpoint protein in regulating G1 to S phase [13]. In previous reports, the effects of berberine were shown to be largely attributed to cell cycle arrest at the G1 phase through the down-regulation of Cyclin D1 [25,26]. Our results are well correlated with these findings when berberine reduced PCNA and cyclin D1 protein expression in MDA-MB-468 to block their proliferation progress in G1 cell cycle phase. On the other hand, using MDA-MB468, Lin, Y.S. et al. [20] demonstrated that berberine treatment at 6 and 12 µM for 48h blocked the cell cycle in G0/G1 with a simultaneous decrease in cyclin D1 expression. However, these results are not in agreement with ours, since the treatment of these cells with berberine for 72 h did not induce any significant cell cycle arrest but it did at 120 h (Figure 3), suggesting that the effect of berberine on cell cycle is dose and time dependent.

Targeting cell cycle progression as a therapeutic intervention has a limited efficacy as it could be only cytostatic [14]. Fortunately, berberine treatment not only caused cell cycle arrest on all treated TNBC cell lines but also triggered apoptosis. Apoptosis includes two major signaling pathways: the extrinsic death receptor pathway and the intrinsic mitochondrial one [27]. To induce apoptosis, several biochemical events occur including the cleavage of caspases as indicators of the intrinsic apoptotic pathway besides of the cleavage of PARP protein. PARP cleavage has been considered as a hallmark of apoptosis caused by DNA strand breaks [9]. In a previous study, it has been reported that no apoptosis was observed after treatment with berberine for 48 h neither against MDA-MB-468 at 12 µM nor toward MDA-MB-231 at 25 µM [20]. However, in our study, no significant apoptosis induction was observed at 72 h and 120 h. Although, when we increased the incubation time to 144 h, we found that berberine significantly induced apoptosis in all studied cells, e.g., MDA-MB-468 at 1 µM, HCC70 at 0.5 and 1 µM, BT-20 at 0.2, 0.5 and 1 µM. At the molecular level, cleaved PARP, cleaved caspase-8 and cleaved caspase-7 proteins expression started earlier at 120h to induce apoptosis in TNBC cells.

Therefore, berberine is also known for its ability to engender apoptosis in several cancer types including breast cancer cells by triggering DNA damage [28]. Moreover, a previous study reported that berberine induces DNA strand breaks in MDA-MB-231 and BT-549 TNBC cell lines at 49.08 µM and 44.58 µM, respectively, in 48h upon the treatment [16]. Some studies suggest that berberine may directly bind to DNA by intercalation [28]. Based on these findings, we led our research to see whether the resulted apoptosis is caused by DNA damage in TNBC cells, by quantifying the amount of P-H2AX expression. H2AX, a variant form of histone H2A, becomes phosphorylated in response to DNA double-strand breaks [29]. Expectedly, we observed a remarkable accumulation of DNA double-strand breaks in berberine-treated cells, as determined by measuring the phosphorylation of H2AX in both HCC70 and BT-20 cells. Inversely, we didn’t notice any effect on the phosphorylation of H2AX in MDA-MB-468 when treated with berberine, suggesting either that berberine induces DNA double-strand breaks depending on the cell line, or either we need to explore the DNA damage in MDA-MB-468 by other techniques in coming perspectives.

Cancer therapy involving cytotoxic drugs kills cells that have a high level of proliferation and regeneration. While this type of therapy targets tumor cells, it affects rapidly proliferating non tumor cells such as skin and hair, accounting for the high level of toxicity associated with such treatments [13].

In our study, we showed that berberine treatment is significantly tolerated by normal human breast epithelial cells MCF-10A when cultured in a 3D matrigel model in comparison to MDA-MB-468 cells cultured in the same conditions. These results are related to some previous studies where it was reported that berberine exhibited no significant effect on the breast normal epithelial cell growth [30], nor in normal human peripheral blood mononuclear cells (PBMC) [31]. Actually, this is important when the systemic toxicity of chemotherapy drugs treatments is the major conventional therapy inconvenient.

The effectiveness of berberine in impairing the growth of TNBC cells without affecting the growth of normal human breast epithelial cells indicates that it may serve as a potential natural compound for TNBC drugs development.

## 4. Materials and Methods

### 4.1. Reagents

Cell culture reagents were all purchased from Life Technologies (Camarillo, CA, USA). All human TNBC and the immortalized breast epithelial MCF10A cell lines were purchased from ATCC (Manassas, VA, USA). Cell lines were authenticated in 2018 and they do not exceed 15 passages in cell culture. Berberine chloride hydrate ≥90% was purchased from Sigma Aldrich (Saint-Louis, MO, USA).

### 4.2. Cell Culture

The TNBC cell line (BT-20) was cultured in MEM Eagle from Sigma Aldrich (Saint-Louis, MO, USA) medium with 10% fetal bovine serum (FBS), 1% glutamax, 1% penicillin/ streptomycin, 1.5 g/L sodium bicarbonate, 0.1 mM non-essential amino-acids, and 1 mM sodium pyruvate.

HCC70, HCC38, HCC1937, HCC1143 and BT-549 cell lines were cultured in RPMI 1640 medium from Fisher Scientific (Waltham, MA, USA) with 10% FBS, 1% glutamax, 1% penicillin/ streptomycin, 1.5 g/L sodium bicarbonate, 10 mM Hepes, and 1 mM sodium pyruvate.

MDA-MB-468 cells were cultured in RPMI 1640 medium with 10% FBS, 1% glutamax, and 1% penicillin/streptomycin.

MDA-MB-231 cells were cultured in DMEM/F12 with 10% FBS and 5% Penicillin/streptomycin.

MCF10A cells were cultured in DMEM/F12 with 5% horse serum, 1% penicillin/streptomycin, 20 ng/mL EGF, 100 ng/mL cholera toxin, 0.01 mg/mL insulin, and 500 ng/mL hydrocortisone.

All the cells were maintained in a humidified 5% CO_2_ incubator at 37 °C.

### 4.3. Assessment of Cell Viability Using MTT Assay

Cell viability was determined by 3-(4,5-dimethylthiazol-2-yl)-2,5 diphenyltetrazolium bromide (MTT) from Sigma-Aldrich (Saint-Louis, MO, USA), a colorimetric assay capable of detecting viable cells by the reduction of the yellow tetrazolium salt to purple formazan. In order to ensure their adhesion and their exponential growth on the day of the treatment, the cells were seeded in 96-well plates at the proper density 2 days (48 h) before the treatment (Table 1). In the day of the treatment, the cells were treated with berberine already dilluted in Dimethylsulfoxyde (DMSO) (Sigma-Aldrich). After the day of the treatment, the cells were incubated for 4 times their doubling time, as described in [32] (Table 2). DMSO (1%), non-treated cells and Doxorubicin were used as negative and positive controls, respectively. At the end of the incubation period, the cells were treated with MTT (5 mg/mL in Phosphate Buffered Saline (PBS)) for 4 h at 37 °C. Subsequently, 10% Sodium Dodecyl Sulfate (SDS) in 10 mM hydrochlorid acid was added to dissolve the formazan, and then the cells were incubated overnight at 37 °C. The production of formazan which is directly proportional to the viable cell number was read as absorbance values at 540 nm using Infinite 200 (Tecan, Lyon, France) with Magellan data analysis software. Experiments were done in triplicate. Cell viability percentage curves and IC_50_ were calculated using Microsoft Excel 2010 from Microsoft Corporation, Microsoft Casablanca (Casablanca, Morocco). The curves were fitted as logarithmic tendency curves then IC_50_ were calculated using their log equations.

### 4.4. Two-Dimensional Clonogenic Assay

Two-dimensional (2D) clonogenic (or colony formation) assay is an *in vitro* cell survival test based on the ability of a single cell to grow into a colony by undergoing multiple divisions [33]. Viable cells were seeded to 6 wells plates (Ø35mm) at 2000 cells/well of MDA-MB-468, at 5000 cells/well of HCC70 and 7000 cells/well of BT-20, and allowed to attach to the well bottom overnight. Cells were treated then with 0.2 µM of berberine, and incubated for 10 days for MDA-MB-468 and for 15 days for both HCC70 and BT-20, time to allow colonies to form in the negative control DMSO (0.005%). The obtained colonies were washed with PBS (1X), fixed and stained with 0.05% of Coomassie^®^ Brilliant Blue R-250 from MPBiomedicals (Aurora, Ohio, USA) solubilized in 50% of methanol, 10% Acetic acid and 40% of Milli-Q water. The colonies number of each line was then compared to DMSO control treated cells using ImageJ software. Experiments were carried out in triplicate and for at least three times.

### 4.5. Cell Cycle Analysis by Flow Cytometry

Cells were seeded to 6 wells plates (Ø35 mm) at 2 × 10^5^ cells/well for the MDA-MB-468 and BT-20 cells, and at 3 × 10^5^ cells/well for HCC70 cells, and allowed to attach to the well bottom overnight.

Cells were treated with berberine at the doses of 0.2, 0.5, and 1 µM, and incubated for 72 and 120 h in 37 °C with 5% CO_2_. DMSO (0.005%) was used as control. After respective incubation times, both attached and detached cells were harvested, washed three times and centrifuged at 1500 rpm (for 5 min) with PBS (1X). The cells were then fixed with 70% of cold ethanol and incubated for 30 min in RT. Next, the cells were washed twice with PBS (1X). After removing ethanol, the pellet was resuspended in PBS containing 10µg/mL of propodium iodide (invitrogen P3566) and 100 µg/mL of RNAase (invitrogen 12091-021). The pellet was then incubated for 15 min at RT. Finally, the cell cycle analysis was carried out with LSRII flow cytometer (Becton Dickinson) using DIVA™ software to determine cellular DNA content. The cell population percentage of each phase was analyzed using FlowJo and ModFit LT software package.

### 4.6. Apoptosis Annexin-V staining

Cells were seeded to 6 wells plates (Ø35 mm) at 2 × 10^5^ cells/well for the MDA-MB-468 and BT-20 cells, and at 3 × 10^5^ cells/well for HCC70 cells, and allowed to attach to the well bottom overnight. Cells were treated with berberine at the doses of 0.2, 0.5 and 1 µM, and incubated for 120 h and 144 h in 37 °C with 5% CO_2_. DMSO (0.005%) was used as control. After incubation, both attached and detached cells were harvested and washed twice by PBS (1X). Next, the cells were then stained with 1% Annexin-V-FITC diluted in 4-(2-hydroxyethyl)-1-piperazine ethane sulfonic (HEPES) buffer for 15 min at RT in the dark. Cells were then washed and stained with 1% propodium iodide diluted in HEPES buffer. The analysis and populations quantification were achieved through LSRII flow cytometer (Becton Dickinson) using DIVA™ and FlowJo softwares.

### 4.7. Molecular Analysis by Western Blotting and Antibodies

Briefly, 1 × 10^6^ of MDA-MB-468 and BT-20 cells, and 1.5 × 10^6^ of HCC70 cells were seeded in (Ø90mm) Petri dishes and allowed to attach overnight, then treated with berberine at 0.5 and 1 µM, and then incubated for 120 and 144 h in 37 °C with 5% CO_2_. DMSO (0.005%) was used as control. For whole cell protein lysates, the samples were prepared according to the established protocol [32]. Briefly, the lysates were collected using the Laemmli 1X buffer. Proteins dosage was done by BCA Protein assay kit reducing Agent Compatible (Perbio, ref 23250). Proteins concentrations were read as absorbance values at 562 nm using Infinite 200 (Tecan, Lyon, France) with Magellan data analysis software. Equivalent amounts of protein lysates (20 µg), were separated by electrophoresis in acrylamide gel (Bio-Rad TGX 4–15%) starting with 15 mA then increasing to 25 mA, then blotted onto nitrocellulose membrane (Bio-Rad, Marnes la Coquette, France). The membranes were then saturated with 5% BSA+TBS/0.1% Tween for 1 h at RT.

The blotted membranes were incubated with different primary antibodies for 2 h at RT, and then washed four times with TBS/0.1% Tween 5 min each, at RT. The membranes were then followed by incubations with secondary antibodies labeled with horseradish peroxidase (Jackson Immuno Research Laboratories, Interchim, Clichy, France) for 1 h at RT.

The proteins were visualized using ECL kit (Amersham Pharmacia Biotech, Orsay, France). The quantification was performed using a LAS-3000 Luminescent Image analyser and Multi Gauge software (Fuji, FSVT, Courbevoie, France). Actin was detected for normalisation between samples using anti-beta-actin primary antibody at the dilution of 1:2000 (Sigma-Aldrich, Saint Quentin Fallavier, France). MGMT, PCNA, cleaved-PARP (cl-PARP), cleaved-caspase-7 (cl-caspase-7) and cleaved-caspase-8 (cl-caspase-8) antibodies were used at 1:1000 dilutions. Cyclin D1 antibody was used at 1:10000 dilution. Histone H2AX and phosphorylated-H2AX (P-H2AX) antibodies were diluted at 1:2000. Cyclin B1 antibody was used at 1:1000 dilution. All the antibodies were purchased from Cell Signaling Technology, Ozyme, Saint Quentin en Yveline, France.

### 4.8. Three-Dimensional Cell Culture

Three-dimensional (3D) cell culture was conducted with matrigel fromBD Biosciences (Le Pont de Claix, France) as described in [10]. Briefly, in 96 well plates, 5200 cell/well of malignant MDA-MB-468 cells and 6500 cell/well of normal Human breast epithelial cells MCF-10A were seeded between two matrigel coats and cell culture medium, for 7 and 10 days, respectively, at 37 °C with 5% CO_2_. The medium was changed and replaced with a new one every 2 or 3 days.

Cells were then treated with berberine at various concentrations (0.5 and 5 µM). DMSO (0.025%), non-treated cells and BI2536 drug [34] were used as negative and positive controls, respectively. Cell viability was determined 3 days after the treatment using the WST1 Cell Proliferation Assay Kit (Millipore) which is based on the enzymatic cleavage of the tetrazolium salt WST1 to formazan by cellular mitochondrial dehydrogenases present in viable cells. The absorbance was measured using a microplate reader (Infinite 200, Tecan, Lyon, France) with Magellan data analysis software at a wavelenght of 450 nm. Experiments were carried out in triplicate.

### 4.9. Statistical Analysis

Data are represented as the mean ± standard deviation (±SD) of three independent experiments. The statistical significance of the obtained results was evaluated by using a one-way analysis of variance ANOVA at * *p* < 0.05, ** *p* < 0.01 and *** *p* < 0.001, using STATA 14.2 software (Lakeway Drive, College Station, TX, USA).

## 5. Conclusions

To summarize, our study showed that berberine inhibited TNBC cell growth by inducing cell cycle arrest and apoptosis. The expression pattern of the protein markers involved in the regulation of cell cycle progression, DNA damage, and apoptosis was correlated to the inhibition of cellular proliferation, suggesting that berberine may be a good candidate in one of the challenges of the global health care system in making cancer treatment more personalized and effective with reduced to no side effects.

## Figures and Tables

**Table 1:** IC_50_ (µM) values of berberine on TNBC cell lines ± standard deviation.

Cell Line	Berberine IC_50_ (µM)
HCC70	0.19 ± 0.06
BT-20	0.23 ± 0.10
MDA-MB-468	0.48 ± 0.25
HCC1143	1.67 ± 0.74
HCC38	3.24 ± 1.05
BT-549	3.44 ± 1.29
HCC1937	2.30 ± 2.09
MDA-MB-231	16.7 ± 2.37

**Table 2:** Seeded cell concentrations per well used in cell viability assay.

Cell Lines	Cell Concentration × 10^3^ (cell/mL) *	Cells Doubling Time (h)
HCC1937	10	45
HCC70	20	50
MDA-MB-468	10	45
HCC38	20	60
BT-20	10	50
HCC1143	10	60
BT-549	5	30
MDA-MB-231	5	25

* 150µL/Well in 96 Wells Plate.
